# Docking, Synthesis and Anticonvulsant Activity of N-substituted Isoindoline-1,3-dione

**Published:** 2017

**Authors:** Maryam Iman, Atefeh Saadabadi, Asghar Davood, Hamed Shafaroodi, Ali Nikbakht, Abdollah Ansari, Masood Abedini

**Affiliations:** a *Chemical* *Injuries Research Center, Baqiyatallah University of Medical Sciences, Tehran, Iran. *; b *Department of Medicinal Chemistry, Pharmaceutical Sciences Branch, Islamic Azad University, Tehran, Iran. *; c *Department of Pharmacology, Pharmaceutical Sciences Branch, Islamic Azad University, Tehran, Iran.*

**Keywords:** Anticonvulsant, Design, Isoindoline, Seizure, Synthesis

## Abstract

A series of compounds related to ameltolide were studied for anticonvulsant potential in the subcutaneous pentylenetetrazol (sc Ptz) test in mice. These compounds were synthesized and characterized by TLC followed by IR and H^1^NMR. *In-vivo* screening data acquired indicate that most of analogs have the ability to protect against PTZ-induced seizure. Phenytoin (PHT) was employed as the reference prototype antiepileptic drug. All compounds exerted their maximal effects 30 min after administration. Out of the 6 compounds, compound 2 at 40 mg/Kg dose is more potent than phenytoin (reference drug) on clonic seizure. Using a model of the open pore of the Na channel, docking study was performed by AutoDock 4.2 program. Docking study has revealed that these compounds are stabilized through at least one hydrogen bond rises from ketone of phthalimide and residue Thr-87 of domain G of sodium channel.

## Introduction

Epilepsy is a devastating neurological disorder, characterized by recurrent spontaneous seizures either in both brain hemispheres (general seizures) or localized in one or more parts of one or both hemispheres (partial seizures) (1). 

The convulsions of approximately 25% of epileptics are inadequately controlled by current clinically available drugs (2). In addition, many patients that achieve seizure control with medications suffer from medication-induced neurotoxicity and numerous side effects including drowsiness, ataxia, gastrointestinal disturbances, gingival hyperplasia, hirsutism, and megaloblastic anemia (3). In recent years, the field of antiepileptic drug (AED) development has become quite dynamic, affording many promising research opportunities, and there is a continuing demand for new anticonvulsant agents as it has not been possible to control every kind of seizure with currently available antiepileptic drugs.

**Scheme 1:**
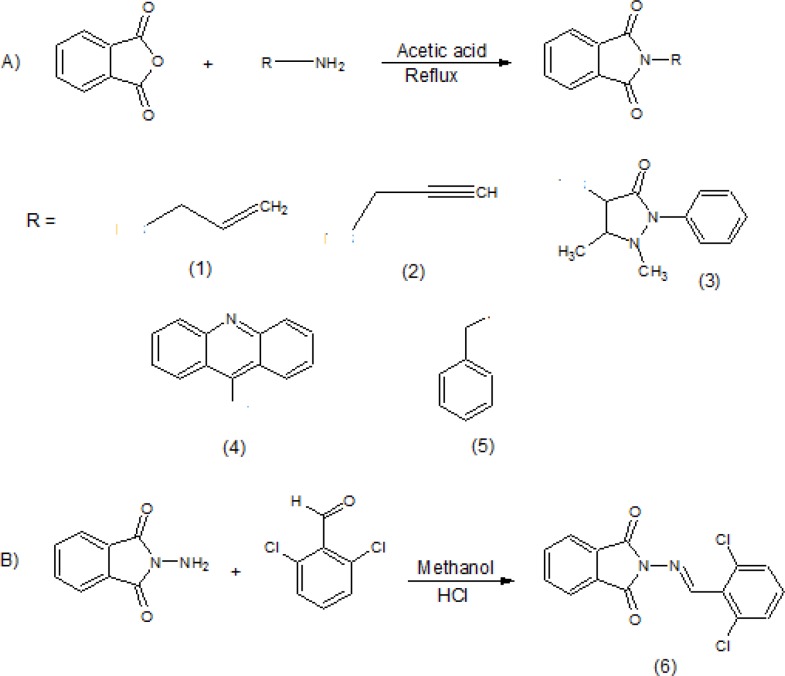
Synthesis of N-substituted phthalimide derivatives

Since voltage-gated sodium (Nav) channels play a critical role in the initiation and propagation of action potentials in excitable cells, they remain a promising target for the development of new AEDs (1). Recently, Phthalimide derivatives were designed based on ameltolide and thalidomide as they possess a similar degree of anticonvulsant potency due to their phenytoin-like profile (4-7). Therefore, as a part of our ongoing research (5-7) in this study a series of phthalimide derivatives were designed and synthesized for having potential anticonvulsant activity (Scheme 1).

## Materials and Methods

*Chemistry*

A group of N-substituted derivatives of the phthalimides (1-6), were synthesized by condensation of the respective amine (aliphatic, aromatic) with phthalic anhydride in acetic acid at reflux temperature (Scheme 1).

All the chemicals and solvents were purchased from Sigma-Aldrich and Merck Company. Thin layer chromatography (TLC) analysis was conducted on Al sheets with a 0.2 mm layer of silica gel (60F254 Merck). Iodine chamber and UV-lamp were used for visualization of TLC spots. Ash less Whatmann No. 1and 2 filter paper was used for vacuum filtration. Melting points (m.p.) were measured in glass capillary tubes using Mel-Temp Laboratory Devices Inc. 

^1^H- and ^13^C-nuclear magnetic resonance (NMR) spectra were measured with Bruker FT-500 spectrometer, and the chemical shifts were reported as parts per million (δ*, *ppm) with (CH_3_)_4_Si (TMS) as an internal standard in DMSO-d6 (for comp. 3 and 4) or CDCl_3_ (for comp. 1, 2, 6 and 5). Signal multiplicities are represented by: s (singlet), bars (broad singlet), d (doublet), t (triplet), m (multiplet). Infrared spectra were acquired on a Nicolet 550-FT spectrometer. Elemental analysis was carried out with a Perkin-Elmer model 240-C apparatus. The results of elemental analysis (C, H, and N) were within 0.4% of the calculated amounts.

*General procedure for preparation of N-substituted phthalimide derivatives (1-5)*

A solution of phthalic anhydride (0.5 g, 3.73 mmol) and appropriate amine (3.73 mmol) in glassial acetic acid (5 mL) was stirred and heated under reflux. The product of this reaction was precipitated by addition of water, filtered, dried, and recrystallized from 95% ethanol to give desired compound (5-7).

*2-allylisoindoline-1, 3-dion (1)*

Using the general procedure and allylamine provided the title compound after 10 h of reflux: White crystals, yield 90%; mp 74.5-76.5 ^o^C (ethanol). 

^1^H-NMR (CDCl_3_): δ 7.877(dd, J = 3.2Hz, J = 5.6Hz, 2H, H_4_, H_7_-phthalimide), 7.738(dd, J=3.2Hz, J=5.6Hz, 2H, H_5_, H_6_-phthalimide), 5.859-5.956(m, 1H, CH), 5.196-5.296(m, 2H, N-CH_2_), 4.305-4.326(m, 2H, CH_2_); ^13^C-NMR(CDCl_3_): δ 168.172(CO), 134.218,132.362, 131.769, 123.555(C=C), 117.997(allylic-CH_2_), 40.293(CH_2_) I; R(KBr) : ν cm^-1^ , 3041(CH-aromatic) , 2922(CH-aliphatic),1822, 1777(CO). Molecular formula: C_11_H_9_NO_2_; Calculated = C(70.58%) H(4.85%) N(7.48%); Found = C(70.63%) H(4.86%) N(7.49%).

*2-(prop-2-ynyl) isoindoline-1, 3-dione (2)*

Using the general procedure and 2-Propynylamine provided the title compound after 8 h of reflux: White crystals, yield 80%; mp 155-156 ^o^C (ethanol).

^1^H-NMR (CDCl_3_): δ 7.902(dd, J = 3Hz, J = 5.39 Hz, 2H, H_4_, H_7_-phthalimide), 7.757(dd, J=3Hz, J=5.4Hz, 2H, H_5_, H_6_-phthalimide), 4.474(d, J=2.4Hz, 2H, CH_2_), 2.239( t, J=2.4Hz, 1H, CH); ^13^C-NMR(CDCl_3_): δ 167.2(CO), 134.5, 132.2, 123.8(C=C), 71.9(C-alkyne), 28.7(CH_2_); IR(KBr): ν cm^-1^ , 3318(CH-alkyne), 3095(CH-aromatic) , 2965(CH-aliphatic), 2650(C-alkyne), 1825, 1780(CO). Molecular formula: C_11_H_7_NO_2_; Calculated = C(71.35%) H(3.81%) N(7.56%); Found = C(71.40%) H(3.81%) N(7.55%).

*2-(2, 3-dihydro-1, 5-dimethyl-3-oxo-2-phenyl-1H-pyrazol-4-yl) isoindoline-1, 3-dione (3)*

Using the general procedure and 4-amino-1,5-dimethyl-2-phenylpyrazolidin-3-one provided the title compound after 12 h of reflux: White crystals, yield 71%; mp 226.4-226.7 ^o^C (ethanol)

^1^H NMR (DMSO-d6) : δ 7.986-7.960(m, 2H, H_4,7_-phthalimdie), 7.937-7.912(m, 2H, H_5,6_-phthalimdie), 7.540(t, J = 7.30 Hz, 2H, H_3_, H_5_-phenyl),7.39(d, J = 7.55Hz, 2H, phenyl), 7.385(t, J = 7.34Hz, 1H, phenyl), 3.243(s, 3H, CH_3_), 2.240 ppm (s, 3H, CH_3_). ^13^C NMR (DMSO-d6): δ 167.684 (CO), 161.491, 154.940, 135.792, 135.330, 132.392, 130.117, 127.902, 125.387 and 124.469 (C=C), 36.112 and 11.247 (CH_3_).IR (KBr): ν cm^-1^, 3058 (CH-aromatic), 2940(CH-aliphatic), 1785, 1725(CO).; Molecular formula: C_19_H_17_N_3_O_3_; Calculated = C(68.05%) H(5.11%) N(12.53%); Found = C(68.01%) H(5.12%) N(12.52%). 

*2-(acridin-9-yl) isoindoline-1, 3-dione (4)*

Using the general procedure and 4-amino-1,5-dimethyl-2-phenylpyrazolidin-3-one provided the title compound after 12 h of reflux: Yellow crystals, yield 55%; mp 258-260 ^o^C (ethanol).

^1^H-NMR (DMSO-d6): δ 8.591(d, J = 8.51 Hz, 2H, H_1, 8_-acridine), 8.183-8.165(m, 2H, H_4, 7_-phthalimide), 7.899-7.855(m, 4H, H_5, 6_-phthalimie and H_4, 5_-acridine), 7.504-7.469(m, 4H, acridine).; IR (KBr): ν cm^-1^, 3133 (CH-aromatic), 1725, 1700(CO). Molecular formula: C_21_H_12_N_2_O_2_; Calculated = C(77.77%) H(3.73%) N(8.64%); Found = C(77.82%) H(3.74%) N(8.65%). 

*2-benzylisoindoline-1, 3-dione (5)*

Using the general procedure and benzylamine provided the title compound after 10 h of reflux: Yellow crystals, yield 55%; mp 115.5-116.5 ^o^C (ethanol).

^1^H NMR (CDCl_3_): δ 7.60-7.89(m, 4H, aromatic), 7.23-7.51(m, 5H, aromatic), 4.48(s, 2H, CH_2_). IR (KBr) : ν cm^-1^, 1716, 1767(CO).; Molecular formula: C_15_H_11_NO_2_; Calculated = C(75.94%) H(4.67%) N(5.90%); Found = C(75.99%) H(4.68%) N(5.91%). 

*2-(2,6-dichlorobenzylideneamino) isoindoline-1, 3-dione (6)*

2,6-dichlorobenzaldehyde (1.23 mmol) was added to a solution of N-aminophthalimide (200 mg, 1.23 mmol) in methanol (7 mL). The PH of the reaction mixture was adjusted between 3 and 4 by addition of concentrated HCl and the reaction mixture was stirred for 2–4 h.The product of this reaction was precipitated by addition of water, filtered, dried, and recrystallized from ethanol to give the titled compound as White crystals, yield 61%; mp 232-234°C (ethanol).

^1^H-NMR (CDCl_3_): δ 9.72(s, 1H, N=CH), 7.73-8.02 (m, 4H, phthalimide), 7.28-7.42 (m, 3H, phenyl). ;IR (KBr): ν cm^-1^ 3062 (H-aromatic), 1767, 1797, (C= O phthalimide), 1721(CH=N).; Molecular formula: C_15_H_8_Cl_2_N_2_O_2_; Calculated = C(56.45%) H(2.53%) N(8.78%) ; Found = C(56.54%) H(2.54%) N(8.79%). 

*Evaluation of the anticonvulsant activity, pentylenetetrazole (PTZ) seizure threshold test*

Adult male albino mice (NMRI strain) weighing 20-30 g and maintained at room temperature (25–30 °C) with 45% humidity were used as experimental animals. The animals were housed in an adequate diet with free access to food and water except during the short time they were removed from their cages for the experimental procedures. All procedures were carried out in accordance with the institutional guidelines for animal care and use.

The ability of the compounds 1–6 to protect against PTZ-induced seizure, tonic and colonic, was determined by an in vivo assay. Each compound was dissolved in DMSO with 2 mg/mL concentration, injected intraperitoneally (i.p.) in groups of 4-6 animals, and screened for anticonvulsant activities at dose levels of 10, 20, and 40 mg/Kg compared with phenytoin as a positive control and a control group of 4 mice were injected just with DMSO at a dose of 0.05 mL/10 g . The single doses of all compounds (40 mg/Kg) were administered 15, 30 or 60 min prior to distinct groups of mice obtained a threshold convulsion. Each animal was placed into an individual plastic cage for observation lasting 1h for tonic-clonic convulsions or death (5).

*Molecular Modeling and Docking Study*

The molecular modeling and docking procedure based on our previous methods have been reported. In short, it is a model of the open pore of the Na channel that was developed based on homology model of the crystal structures of K channel (8-13). The chemical structures of inhibitors (scheme 1) were constructed using HyperChem software (version 7, Hypercube Inc.). Conformational analysis of the favorite compounds was executed through Semi-empirical molecular orbital calculations (PM3) method by utilization of the HyperChem software. Docking calculations were exerted using AutoDock 4.2 software (14-16) and a model of the open pore of the Na channel was utilized as a receptor (17). The implementing Lamarckian Genetic Algorithm (LGA) was adopted to perform the molecular docking studies. Final docked conformations were clustered using a tolerance of 1 A˚ root mean square deviation (RMSD) and the docking log (dlg) files were analyzed using the AutoDock Tools (version 1.5.6). 

## Results and Discussion

*Chemistry*

Six new derivatives of Phthalimide pharmacophore were synthesized in 55- 95% yield based on the method that is shown in Scheme 1. All the compounds were characterized by TLC followed by IR and NMR (Table 1).

*Anticonvulsant activity screening*

**Figure 1:**
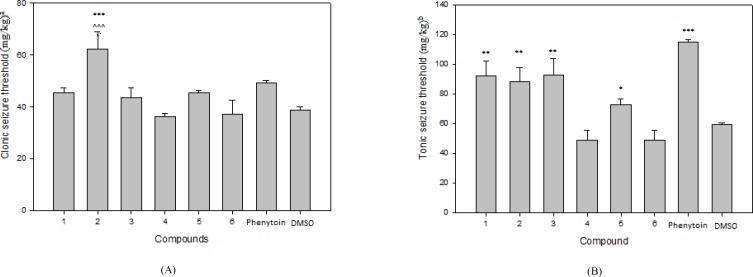
Effect of phenytoin and compounds 1-6 on clonic (A) and tonic (B) seizure threshold induced by PTZ in mice. Animal received vehicle or drugs (40 mg/Kg), 30 min before PTZ administration. Data are expressed as mean ± S.E.M. * P < 0.05, **P < 0.01, ***P < 0.001 compared to vehicle and ^ ^ ^ *P *< 0.001 compare to phenytoin

**Figure 2:**
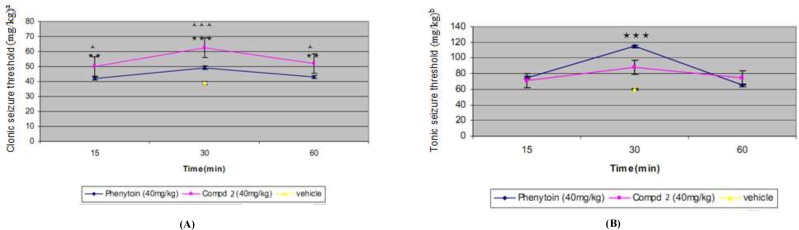
The time course of the effects of phenytoin (40 mg/Kg) and compound 2 (40 mg/Kg) on clonic (A) and tonic (B) seizure threshold by PTZ. Phenytoin or Compound 5 was administered 15, 30 and 60 min before PTZ and their effect compared to vehicle (30 min before test). Data are expressed as mean ± S.E.M. **P < 0.01, *** *P *< 0.001 compared to vehicle group (30 min) and ^ * P* < 0.05, ^ ^ ^ * P *< 0.001 compare to phenytoin

**Figure 3:**
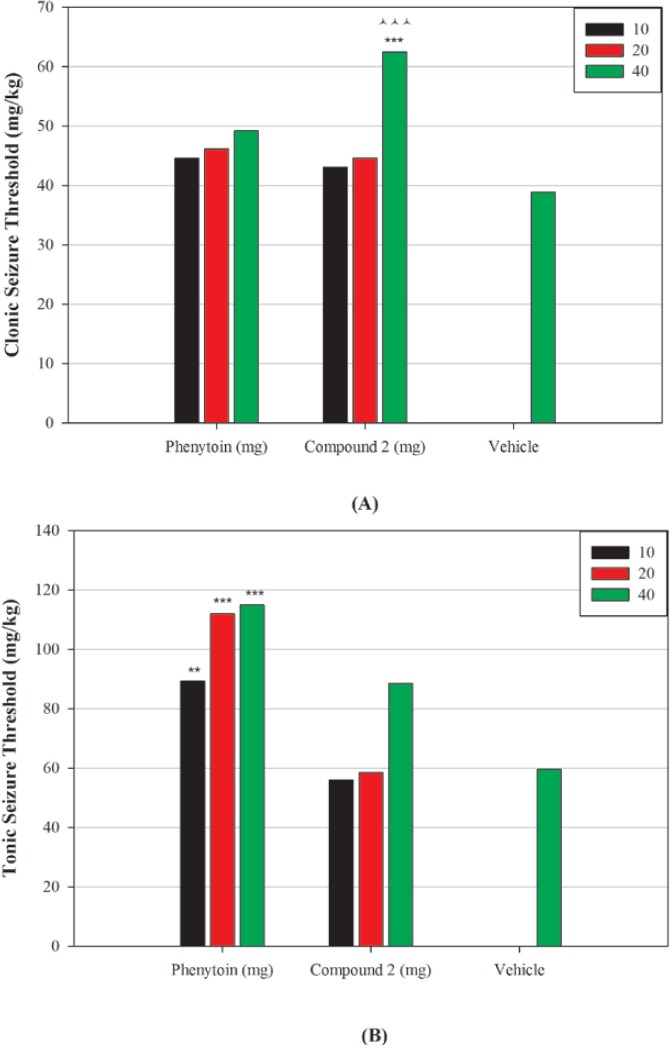
Effect of different doses of phenytoin and compound 2 on clonic (A) and tonic (B) seizure threshold induced by PTZ in mice. Animal received vehicle or drugs (10, 20, 40 mg/Kg), 30 min before PTZ administration. Data are expressed as mean ± S.E.M. **P < 0.01, ***P < 0.001 compared to vehicle, ^ ^ ^ * P *< 0. 001 compared to corresponding phenytoin group

The results of pharmacological evaluation (Table 2) demonstrate that except compounds 4 and 6 all derivatives have the ability to protect against PTZ-induced seizure (Figure 1). The maximum effects of all compounds and phenytoin were revealed at 30 min after administration (Figure 2). Compounds 1, 2, 3 and 5 elevated the colonic and tonic seizure thresholds at 30 min that only compounds 1 and 2 at dose of 40 mg/Kg showed anticonvulsant activity on clonic seizure significantly (Figure 1). Compound 2 at 40 mg/Kg dose is more potent than phenytoin (reference drug) on clonic seizure (Figure 3). The pharmacological evaluation reveals that compounds 3, 1, 2 and 5 are most effective on the tonic seizure thresholds respectively (Figure 1).

**Figure 4:**
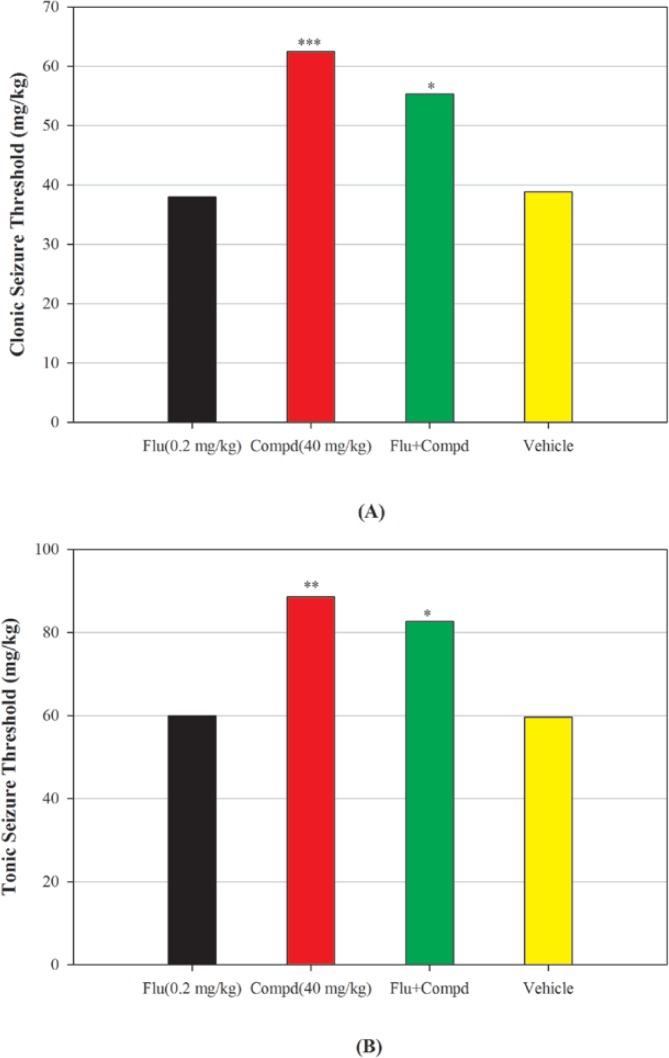
Effect of benzodiazepine receptor antagonist flumazenil (0.2 mg/Kg) on anticonvulsant effect of compound 2, (A): clonic seizure threshold and (B) tonic seizure threshold. Flumazenil was administered 15 min before compound 2 (40 mg/Kg) or its vehicle and 45 min before PTZ. Data are expressed as mean ± S.E.M. *P < 0.05, ***P < 0.001 compared to vehicle

Blockade of benzodiazepine receptor by flumazenil, partially inhibited the anticonvulsant effects of compound 2 on both clonic and tonic PTZ-induced seizures (Figure 4), showing probably GABA-A receptor mediate as well as Na channels in these effects.

**Figure 5:**
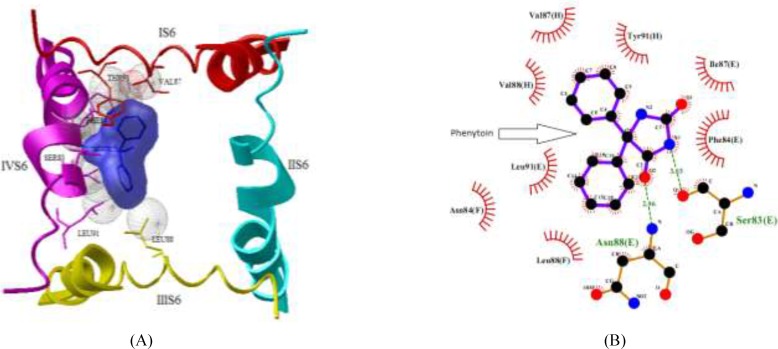
NaV1.2 channel and phenytoin interaction. (a) Cartoon diagram of the channel- drug interactions. (b) Ligplot (18) diagram of the Nav1.2- phenytoin drug interactions. Hydrogen bonds are depicted in green color dotted lines and hydrophobic interaction residues are shown in red color

**Figure 6:**
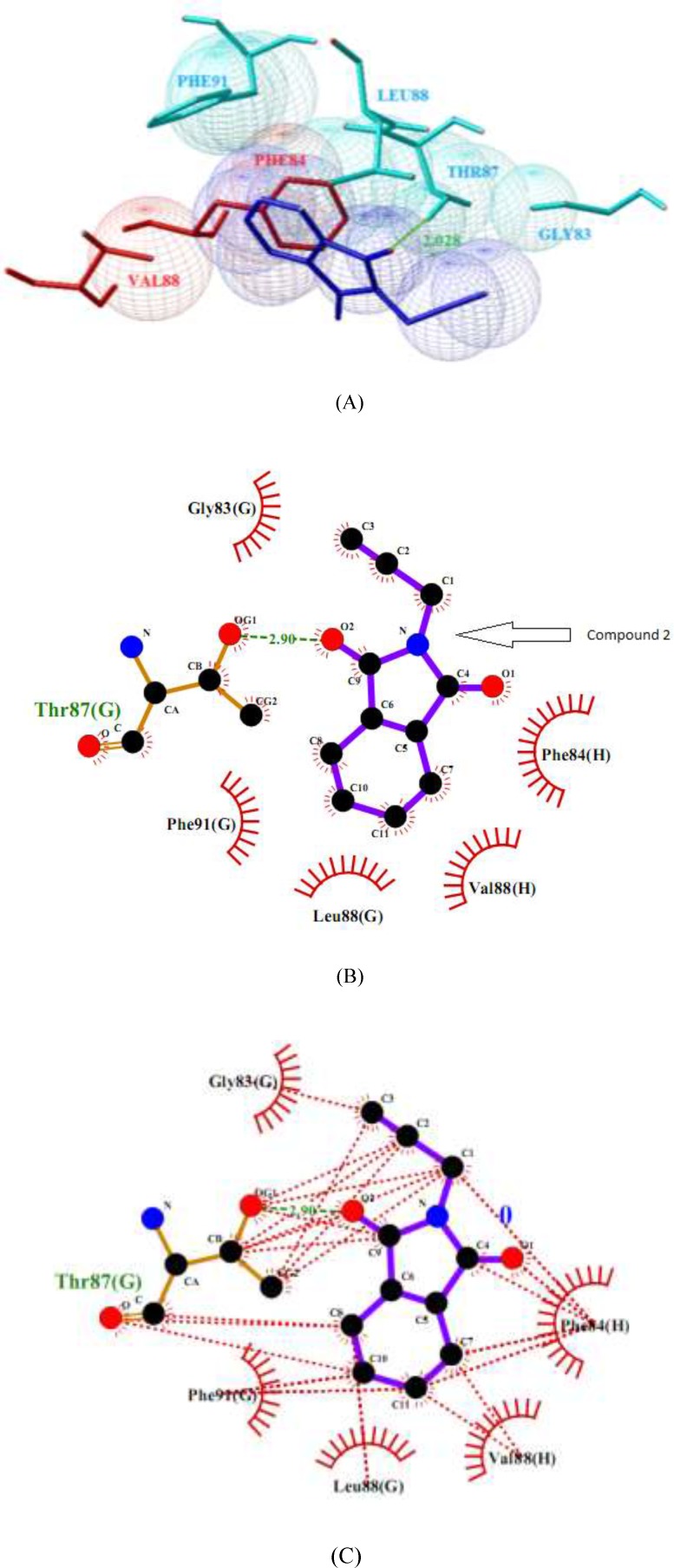
NaV1.2 channel and compound 2 interaction. (a) Cartoon diagram of the channel- ligand interactions. (b and c) Ligplot diagram of the Nav1.2- compound 2 interactions. Hydrogen bonds are depicted in green color dotted lines and hydrophobic interaction residues are shown in red color

**Table 1:** Physical properties of the tested compounds (1–6)

**compound**	**Mol. formula**	**Mol. Weight**	**m.p (** ^ο^ **C)**	**Yield%**	**Log p** a
1	C_11_H_9_NO_2_	187.19	74.5-76.5	90	1.73
2	C_11_H_7_NO_2_	185.18	155-156	80	1.26
3	C_19_H_17_N_3_O_3_	335.36	226.4-226.7	71	2.79
4	C_21_H_12_N_2_O_2_	324.33	258-260	55	4.16
5	C_15_H_11_NO_2_	237.25	115.5-116.5	55	2.77
6	C_15_H_8_ Cl_2_N_2_O_2_	319.14	232-234	61	2.14

a Log p was calculated using HyperChem software.

**Table 2:** Anticonvulsant activity of the examined compounds (1–6) in mice at dose 40 mg/Kg.

**Compounds**	**CST** a	**TST** b
1	45.61 ± 1.69	92.096 ± 10.04
2	62.46 ± 6.48	88.6 ± 9.02
3	43.52 ± 3.72	92.76 ± 10.99
4	37.30 ± 5.29	48.64 ± 6.67
5	45.48 ± 0.85	72.63 ± 3.98
6	36.1 ± 1.27	48.64 ± 6.67
Phenytoin	49.20 ± 1.09	115.00 ±1.85
Vehicle(DMSO)	38.9 ± 1.01	59.58 ±1.012

a clonic seizure threshold

b tonic seizure threshold

**Table 3:** Docking result of ameltolide analogues by using of Auto dock software (version 4)

**No. com**	**Binding energy ** a	**K**_i_ ** (µM)** b	**Intermol energy ** c	**Electrostatic energy**	**Total internal energy**	**Torsional energy**	**Unbound energy**	**Log P**	**Docking energy** d
1	-4.81	298.91	-5.4	-0.05	-0.17	0.6	-0.17	1.73	-5.57
2	-4.97	227.42	-5.27	-0.03	-0.1	0.3	-0.1	1.26	-5.37
3	-7.18	5.45	-7.78	-0.07	-1.15	0.6	-1.15	2.79	-8.93
4	-6.86	9.42	-7.15	0.01	-0.63	0.3	-0.63	4.16	-7.78
5	-5.7	66.54	-6.29	-0.04	-0.37	0.6	-0.37	2.77	-6.66
6	-6.7	12.27	-7.3	-0.01	-0.03	0.6	-0.03	2.14	-7.33
PHE	-5.83	53.37	-6.43	-0.04	-0.71	0.60	-0.71	2.08	-6.74

PHE: Phenytoin(reference drug)

a The predicted binding energy (Kcal/mol) is the sum of intermolecular energy and Torsional free energy.

b Inhibition constant (K_i_): exp (∆G×1000) / (R_cal_×TK) where ∆G is the docking energy, R_cal_is 1.98719 and TK is 298.15.

c Intermolecular energy is sum of Vdw-hb-desolv-energy and Electrostatic-energy.

d Docking energy is the sum of intermolecular energy and ligand^’^s internal energy

*Molecular Modeling and Docking Results*

Based on the procedure explained in the experimental section, the predicted binding energy, docked energy, and inhibition-constant (Ki) of these inhibitors into the active site are listed in Table 3. 

Molecule phenytoin is comfortably occupied at the domain IV-S6 of NaV1.2 via two strong hydrogen bonds using hydantoin ring of phenytoin and the residues of Ser-83 and domain E Asn-88 of sodium channel (Figure 5). In addition, some hydrophobic interactions by the residues Phe-84, Ile-87 and Leu-91 of domain E, Asn-84 and Leu-88 of domain F and Val-87, Val-88 and Tyr-91 of domain H also help the ligand molecule for its conformational stability. The NaV1.2 channel and compound 2 complex molecules (Figure 6) are stabilized through one hydrogen bond rises from ketone of phthalimide and residue Thr-87 of domain G of sodium channel. In addition, some hydrophobic interactions by the residues Gly-83, Phe-91 and Leu-88 of domain G and residues Val-88 and Phe-84 of domain H are observed. 

The main idea of designing of these compounds was based on Clark and Coworkers researches that finally N-phenyl phthalimide pharmacophore was created from a combination of the models of ameltolide and thalidomide that exhibited phenytoin-like profile (17, 19-25). The research on N-phenyl ring of this pharmacophore was continued and new derivatives were designed as anticonvulsant agents. Interactions of anticonvulsant agents with the voltage-gated sodium channels have been previously documented for N-phenyl derivatives and phenytoin (26). In this study, we applied docking analysis to clarify the drug-receptor interactions for showing better structure–activity relationships. Anticonvulsant activity of these compounds determined with PTZ induced seizure, tonic and colonic *in-vi*vo assay. Generally, our results are in line with previous studies so that compound 2 was more potent than phenytoin in clonic seizure, but compound 4 and 6 did not show any anticonvulsant effect compared to vehicle. In conformity with docking study these two compounds have not formed any hydrogen bond with Tyr87 of domain II. So, lack of anticonvulsant effect might be because of low affinity compound 4 and 6 to the receptor.

## Conclusion

Six analogs of phthalimide derivatives were synthesized, characterized by TLC followed by IR and proton NMR and also their ability to protect against pentylenetetrazole-induced seizure *in-vivo* was investigated in mice. Based on our *in-vivo *screening data, four compounds of the synthesized analogs have the ability to protect against pentylenetetrazole-induced seizure. These compounds exerted their maximal effects 30 min after administration. The most potent compound in clonic seizure was compound 2, which was more active than the reference drug phenytoin. Also, the docking analysis have showed a hydrogen binding interaction formed between compound 1,2,3 and 5 and receptor. In conformity docking study and pharmacological data, it can be concluded the hydrogen binding interaction has an important role in inhibiting of sodium channel receptor.

Thus, the present study has demonstrated that new compounds can be synthesized, being able to make hydrogen binding interaction with drug receptor.
